# Supplementary material for: Development of a predictive model for severe hyperlipidemic acute pancreatitis based on LASSO regression: a retrospective study
Source: PeerJ. 2025 Dec 5;13:e20471. doi: 10.7717/peerj.20471 (PMC12684402; doi:10.7717/peerj.20471)
Supplement: Supplemental Information 3 [file peerj-13-20471-s003.docx]

Codebook

Sex 1=male，2=female

fever 0=No，1=Yes

mindchange 0=No，1=Yes

fattyliver 0=No，1=Yes

diabetes 0=No，1=Yes

renalfailure 0=No，1=Yes

septicemmia 0=No，1=Yes

replase 0=No，1=Yes

UGLU 0=No，1=Yes

urineketone 0=No，1=Yes

urineprotein 0=No，1=Yes

type 0=mild，1=moderate severe/severe
